# Bioimage classification with subcategory discriminant transform of high dimensional visual descriptors

**DOI:** 10.1186/s12859-016-1318-9

**Published:** 2016-11-16

**Authors:** Yang Song, Weidong Cai, Heng Huang, Dagan Feng, Yue Wang, Mei Chen

**Affiliations:** 1 School of Information Technologies, The University of Sydney, Sydney, Australia; 2 Department of Computer Science and Engineering, University of Texas, Arlington, USA; 3 Med-X Research Institute, Shanghai Jiaotong University, Shanghai, China; 4 Bradley Department of Electrical and Computer Engineering, Virginia Polytechnic Institute and State University, Arlington, USA; 5 Computer Engineering Department, University of Albany State University of New York, Albany, USA; 6 Robotics Institute, Carnegie Mellon University, Pittsburgh, USA

**Keywords:** Microscopy imaging, Classification, Subcategory model, Discriminative feature transform

## Abstract

**Background:**

Bioimage classification is a fundamental problem for many important biological studies that require accurate cell phenotype recognition, subcellular localization, and histopathological classification. In this paper, we present a new bioimage classification method that can be generally applicable to a wide variety of classification problems. We propose to use a high-dimensional multi-modal descriptor that combines multiple texture features. We also design a novel subcategory discriminant transform (SDT) algorithm to further enhance the discriminative power of descriptors by learning convolution kernels to reduce the within-class variation and increase the between-class difference.

**Results:**

We evaluate our method on eight different bioimage classification tasks using the publicly available IICBU 2008 database. Each task comprises a separate dataset, and the collection represents typical subcellular, cellular, and tissue level classification problems. Our method demonstrates improved classification accuracy (0.9 to 9%) on six tasks when compared to state-of-the-art approaches. We also find that SDT outperforms the well-known dimension reduction techniques, with for example 0.2 to 13% improvement over linear discriminant analysis.

**Conclusions:**

We present a general bioimage classification method, which comprises a highly descriptive visual feature representation and a learning-based discriminative feature transformation algorithm. Our evaluation on the IICBU 2008 database demonstrates improved performance over the state-of-the-art for six different classification tasks.

## Background

Bioimage informatics has become an increasingly important field in recent years owing to the advances in microscopy imaging technologies [1, 2]. Automated analysis of microscopy images helps to achieve objective and consistent cell phenotype recognition, subcellular localization, and histopathological classification, which are critical to many biological studies [3–8]. In particular, classification of biological images is an essential algorithmic component in such computed-aided analyses.

Existing bioimage classification methods typically comprise two main components: feature extraction and classification. The descriptiveness of feature descriptors is essential to the bioimage classification performance, and much work has been conducted for bioimage feature extraction. The feature descriptors are often multi-modal, i.e. integrating multiple different types of features. The commonly used features include the traditional approaches such as Gabor filters and Haralick textures, and more recent descriptors such as the scale-invariant feature transform (SIFT) and local binary patterns (LBP) [9–21]. To further enhance the discriminative power of feature descriptors for specific problem domains, customized features have been designed manually [22–28] or learned automatically [29–32].

With these extracted descriptors, supervised classification models such as support vector machine (SVM) [12, 13, 18, 19, 22–24, 27, 29, 31], subspace learning [10, 11, 14–16, 26], multiple instance learning [17, 25] and sparse representation [21, 32] are applied. However, the classification performance is often largely affected by the small number of training data available for bioimage research. For example, in the IICBU 2008 database [4], each classification task contains only hundreds of images categorized into multiple classes. The small amount of training data would not well represent the feature space characteristics, and the trained classifier could easily overfit especially with high-dimensional descriptors. The applicability and effectiveness of automated feature learning algorithms would be limited as well. Dimension reduction techniques are sometimes used in bioimage classification to avoid the over-fitting problem [18, 22, 24]. However, the effect of applying these techniques is often not evaluated and the result could actually be negatively affected due to undesirable information loss.

In the broader computer vision field, high-dimensional descriptors, such as the improved Fisher vector (IFV) [33], have become increasingly popular. IFV can be considered as a variation of the bag-of-words (BOW) encoding of local descriptors. While BOW assigns the descriptors to a pool of visual words and computes the histogram distribution of the visual words, IFV formulates Gaussian mixture models from the descriptors and computes the first- and second-order statistics for each feature dimension. With the original IFV, the local descriptors used are dense SIFT features, and the resultant feature dimension of IFV is much higher than BOW. IFV has shown excellent discriminative performance for face recognition, object detection and texture classification [33–35].

In addition, descriptors that incorporate spatial pooling of local descriptors (e.g. LBP [36], histogram of oriented gradients (HOG) [37], GIST [38] and census transform histogram (CENTRIST) [39]) can have high dimensions as well. With spatial pooling, an image is partitioned into a grid or hierarchy of regions and descriptors computed at the region-level are concatenated to form a long overall descriptor, so that the overall descriptor captures the spatial structure of the image to some extent. Recent studies show that high-dimensional descriptors often provide more discriminative power than the lower-dimensional counterparts [40–42].

With high-dimensional features, especially IFV, the linear-kernel SVM classifier is often found to be the most effective and efficient way of training and classification [33, 34]. Furthermore, it has been reported that better classification results can be achieved with properly designed dimension reduction techniques [42–45]. For example, a discriminative metric learning method has been proposed to reduce the dimension of IFV features and improve the classification performance for face images [43, 45]. Dictionary learning methods have also been used to enhance the discriminative power of feature representations. Examples include the Fisher discrimination dictionary learning (FDDL) [46], and latent dictionary learning (LDL) [47]. They can also be used for dimensionality reduction when the dictionary size is smaller than the descriptor’s dimension.

In this study, we propose a new bioimage classification method. We have two main methodological contributions. First, we find that by combining IFV (computed based on local SIFT features) with LBP, HOG, CENTRIST and GIST texture features, the resultant high-dimensional multi-modal descriptor is highly discriminative for a wide variety of microscopy images and classification objectives at the subcellular, cellular, and tissue levels. While IFV has recently been adopted into bioimage classification [27, 31], these approaches are designed for specific problem domains, i.e. HEp-2 cell classification and ovarian cancer classification, whereas our design is validated on a number of classification tasks and we identify a set of texture features that provide complementary information to IFV.

Second, to further enhance the discriminative power of the descriptors, we design a subcategory discriminant transform (SDT) method to transform the descriptors before performing classification. SDT has a similar learning objective to linear discriminant analysis (LDA) in that the descriptor transform is aimed to minimize the within-class variation and maximize the between-class difference. However, SDT performs feature transformation with learned convolution kernels without dimensionality reduction, and the learning objective is modelled based on subcategories rather than the class-level information. We also compare SDT with the popular discriminative dimension reduction (LDA, generalized discriminant analysis (GDA) [48], full matrix learning (FML) [43]), and dictionary learning (FDDL) techniques, and show consistent advantage over them.

**Fig. 1:**
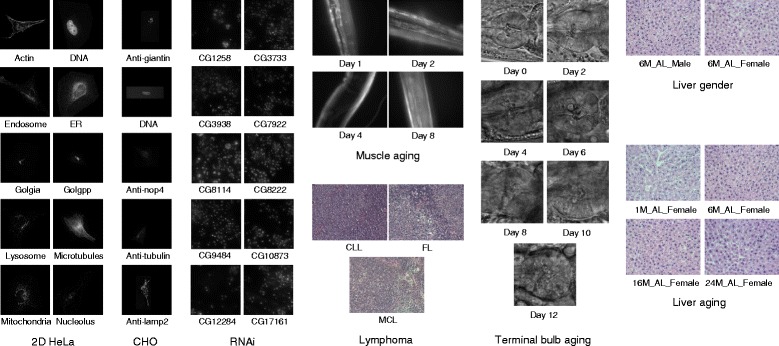
Sample images of the IICBU database. Eight datasets from the IICBU 2008 database are used in this study, including the 2D HeLa, CHO, RNAi, muscle aging, terminal bulb aging, lymphoma, liver gender, and liver aging datasets. Each image represents one image class, and the class name is annotated under the image. Summary of the dataset properties is listed in Table 1. Comprehensive description of the database can be found in [4]

For evaluation, we use the publicly available IICBU 2008 database [4] to perform eight different multi-class classification tasks in the areas of subcellular localization, phenotype recognition and histopathological classification. Figure 1 shows the sample images. We achieve improved classification performance over the current state-of-the-art for six tasks.

**Table 1:** Summary of the IICBU 2008 database used

Dataset	# images	# classes	Microscopy	Description
2D HeLa	860	10	Fluorescence	HeLa cells with 10 stains
CHO	340	5	Fluorescence	CHO cells with 5 stains
RNAi	200	10	Fluorescence	Fly cells of 10 genes
Muscle aging	252	4	Fluorescence	*C.elegans* of 4 ages
Terminal bulb aging	970	7	DIC	*C.elegans* of 7 ages
Lymphoma	375	3	Brightfield	Malignant lymphoma of 3 subtypes
Liver gender	522	2	Brightfield	6-month mice on AL diet of 2 genders
Liver aging	850	4	Brightfield	Female mice on AL diet of 4 ages

DIC: Differential Inference Contrast; CHO: Chinese Hamster Ovary; AL: Ad-libitum

## Methods

**Fig. 2:**
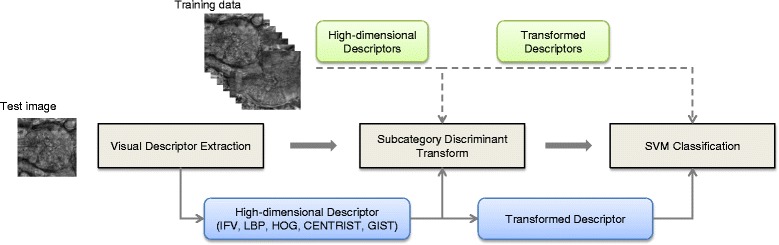
Illustration of our method design. During the training process, the high-dimensional visual descriptors of the training images are extracted, and the SDT models are learned. The visual descriptors of the training images are then transformed using the learned SDT models, and a linear-kernel SVM is learned from the transformed descriptors. During the testing process, the high-dimensional visual descriptor of the test image is extracted, and then transformed using the learned SDT model. The transformed descriptor is then classified using the learned SVM to produce the classification output

Given a set of training images, we first extract the visual descriptors of each image. The kernels of SDT are learned from this training set and the visual descriptors are transformed with these kernels. A multi-class linear-kernel SVM model is then learned based on the transformed visual descriptors. To classify a test image, we extract its visual descriptor, then perform SDT with the learned kernels, and finally obtain its class label with the learned SVM model. Figure 2 illustrates the overall design of our method.

### Visual descriptor extraction

To represent the biological images, we propose to use a combination of five visual descriptors: IFV, LBP, HOG, GIST and CENTRIST. They describe different aspects of the visual characteristics, and we expect their combination to give a more comprehensive image description compared to the individual descriptors. While LBP and HOG are often used in bioimage studies [15, 16, 23], the other descriptors have rarely been used in this field.

The IFV descriptor computes the image-level statistics of a dense set of local SIFT descriptors with Fisher encoding. Specifically, local SIFT descriptors are extracted densely at multiple scales with the width of the SIFT spatial bins set to 2, 4, 6, 8 and 10 pixels and sampled every two pixels. These 128-dimensional SIFT descriptors are reduced to 64 dimensions using PCA and a Gaussian mixture model (GMM) with 64 Gaussian components built to obtain the Fisher encoding. This process produces a 64×64×2 dimensional IFV descriptor.

To compute the LBP, HOG and GIST descriptors, an image is divided into 4×4 grid of cells. For each cell, a 58-dimensional LBP (with 3×3 pixels neighborhood and uniform quantization), 31-dimensional HOG (with directed and undirected gradients of 9 orientations), and 32-dimensional GIST (with 8 orientations and 4 scales) descriptors are extracted. Consequently, the LBP, HOG and GIST descriptors are of 58×16, 31×16 and 32×16 dimensions, respectively. These descriptors encode the texture and shape features with spatial information provided by the cell subdivision. In addition, a 256-dimensional CENTRIST descriptor is extracted globally for the whole image, and is used to capture the overall structural information. Each descriptor is also L2 normalized.

*Block structure of descriptors*. We note that each feature descriptor can be partitioned into multiple blocks of feature vectors. For example, the IFV descriptor can be partitioned into 64×2=128 numbers of 64-dimensional (difference) vectors. The LBP, HOG and GIST descriptors can be partitioned by the cells into 16 vectors of 58, 31 or 32 dimensions, respectively. The histogram-based CENTRIST descriptor can be artificially partitioned into four 64-dimensional vectors. Therefore, the descriptor *x* can be rewritten as *x*={*x*_*b*_:*b*=1,…,*B*}, meaning that *x* is constructed by concatenating *B*=128+16×3+4=180 feature vectors and each vector $x_{b}\in \mathbb {R}^{C\times 1}$ is of *C*= 64, 58, 31, 32 or 64 dimensions. This block-wise representation of descriptors is useful in our proposed SDT algorithm as described in the next section.

### Subcategory discriminant transform

**Fig. 3:**
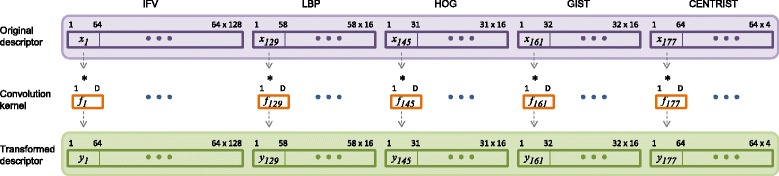
Illustration of the visual descriptor and subcategory discriminant transform. A total of 180 convolution kernels are learned with each kernel corresponding to one block of feature vector within the high-dimensional visual descriptor

While the visual descriptors can be directly used to train a classification model and classify the test images, we design a SDT algorithm to further enhance the discriminative power of the visual descriptors. Formally, let $x\in \mathbb {R}^{H\times 1}$ denote the visual descriptor of an image with *H* dimensions. The objective of SDT is to transform *x* to a more discriminative descriptor $y\in \mathbb {R}^{H\times 1}$. To do this, for each feature vector *x*_*b*_, we compute a transformed vector *y*_*b*_=*x*_*b*_∗*f*_*b*_ with ∗ as the convolution operator and *f*_*b*_ as the convolution kernel, and *y*_*b*_ has the same dimension as *x*_*b*_. Then the transformed descriptor is the concatenation of *B* transformed vectors, i.e. *y*={*y*_*b*_:*b*=1,…,*B*}. Figure 3 illustrates our design of the SDT algorithm.

We design a subcategory-based discriminative learning model to derive the convolution kernels {*f*_*b*_:*b*=1,…,*B*}. Specifically, a kernel is defined as an *D*-dimensional vector $f_{b}\in \mathbb {R}^{D\times 1}$, *D*<*C*. The kernel is supposed to reduce the within-class feature variation and increase the between-class feature difference, so that the transformed vector *y*_*b*_ is more discriminative. In other words, we consider that generally (1) images of the same class would show inhomogeneous visual features and can be grouped into subcategories, and (2) images of different classes could show similar visual features and the subcategories from different classes could overlap in the feature space. By transforming *x*_*b*_ into *y*_*b*_, we expect that (1) the subcategories of the same class are closer hence the within-class variation is reduced, and (2) the subcategories between different classes are more separated hence the between-class difference is increased.

Formally, we denote a set of *N* training images as {*x*(*n*):*n*=1,…,*N*} with *n* as the index of the training images. We first cluster the training images into a set of subcategories. Each subcategory contains images from the same class and exhibiting similar visual descriptors. The subcategories are denoted as {*S*_*l*,*k*_:*l*=1,…,*L*,*k*=1,…,*K*_*l*_}, and a subcategory *S*_*l*,*k*_ is identified by its class label *l* and index *k* within the class *l*, with *L* denoting the number of classes and *K*_*l*_ denoting the number of subcategories in class *l*.

The subcategories are generated by first clustering all training data {*x*(*n*):*n*=1,…,*N*} into *L* clusters regardless of the class labels using the locality constrained subspace clustering (LSC) method [35]. The method first constructs an affinity matrix using the locality-constrained linear coding (LLC) [49], then spectral clustering is performed on the affinity matrix to obtain the clusters. This LSC method is essentially similar to the popular sparse subspace clustering (SSC) [50], but LLC is used in place of the standard sparse representation for efficient computation of the affinity matrix. In our study, we set the sparsity constant to 20 and the balance parameter to 1*e*−4 for all datasets. Then, for each cluster, the data belonging to the same class are extracted as a subcategory. In this way, we avoid choosing the number of subcategories for each class, but just use *L* (i.e. the number of classes) for clustering. Note that because of the large within-class variation and small between-class difference, each cluster typically contains data from a number of classes and the number of subcategories for each class is close to *L*.

Based on the subcategories, we define the within-class variation $V_{with}^{b}\phantom {\dot {i}\!}$ (a scalar) and between-class difference $V_{betw}^{b}\phantom {\dot {i}\!}$ (a scalar) for the transformed feature vectors {*y*_*b*_(*n*):*n*=1,…,*N*} as:

1 $$ V_{with}^{b} = \sum_{l=1}^{L} \sum_{k=1}^{K_{l}}\frac{|S_{l,k}|}{K_{l}-1} \sum_{k'=1}^{K_{l}}\left(\theta_{l,k}^{b}-\theta_{l,k'}^{b}\right)^{2}  $$

2 $$ {}V_{betw}^{b} = \sum_{l=1}^{L}\sum_{k=1}^{K_{l}} \frac{|S_{l,k}|}{\sum_{l'=1}^{L}K_{l'}} \sum_{l'=1}^{L} \sum_{k'=1}^{K_{l'}} \left(\theta_{l,k}^{b}-\theta_{l',k'}^{b}\right)^{2}, \;\; l'\neq l  $$

where |*S*_*l*,*k*_| is the number of descriptors in the subcategory *S*_*l*,*k*_. $\theta _{l,k}^{b}$ is the mean element (a scalar) of the transformed vectors derived from the subcategory *S*_*l*,*k*_:

3 $$ \theta_{l,k}^{b} = \frac{1}{C|S_{l,k}|}\sum_{m=1}^{|S_{l,k}|} \sum_{i=1}^{C}y_{b,i}(m)  $$

where *i* indexes the feature elements in the *C*-dimensional vector *y*_*b*_(*n*), and *m* indexes the descriptors in the subcategory *S*_*l*,*k*_.

Recall that the transformation is computed by *y*_*b*_=*x*_*b*_∗*f*_*b*_. From an element-wise perspective, it can be considered that the individual element *x*_*b*,*i*_∈*x*_*b*_ is transformed to $y_{b,i}=h_{b,i}^{T}J_{D}f_{b}\phantom {\dot {i}\!}$, where $h_{b,i}\in \mathbb {R}^{D\times 1}$ is the vector of length *D* extracted from *x*_*b*_ with its center at the *i*th element of *x*_*b*_. *J*_*D*_ is a *D*×*D* dimensional exchange matrix, so that *f*_*b*_ is flipped vertically for computing the convolution. The mean element $\theta _{l,k}^{b}$ is then equivalent to:

4 $$ \theta_{l,k}^{b} = \frac{1}{C|S_{l,k}|}\sum_{m=1}^{|S_{l,k}|} \sum_{i=1}^{C}h_{b,i}^{T}(m)J_{D}\,f_{b}  $$

For simpler notation, we rewrite Eq. (4) as:

5 $$ \theta_{l,k}^{b} = \gamma_{l,k}^{b}\,f_{b},\;\; \gamma_{l,k}^{b}=\frac{1}{C|S_{l,k}|}\sum_{m=1}^{|S_{l,k}|} \sum_{i=1}^{C}h_{b,i}^{T}(m)J_{D}  $$

with $\gamma _{l,k}^{b}\in \mathbb {R}^{1\times D}$. By substituting Eq. (5) into Eqs. (1) and (2), we obtain:

6 $$ \begin{aligned} {}V_{with}^{b} = & {f_{b}^{T}}\left\lbrace\sum_{l=1}^{L} \sum_{k=1}^{K_{l}}\frac{|S_{l,k}|}{K_{l}-1} \sum_{k'=1}^{K_{l}}\left(\gamma_{l,k}^{b}-\gamma_{l,k'}^{b}\right)^{T} \left(\gamma_{l,k}^{b}-\gamma_{l,k'}^{b}\right)\right\rbrace f_{b} \end{aligned}  $$

7 $$ \begin{aligned} {}V_{betw}^{b} \!= & {f_{b}^{T}}\!\left\lbrace\!\sum_{l=1}^{L}\sum_{k=1}^{K_{l}} \frac{|S_{l,k}|}{\sum_{l'=1}^{L}K_{l'}} \!\sum_{l'=1}^{L} \!\sum_{k'=1}^{K_{l'}} \!\!\left(\!\gamma_{l,k}^{b}\,-\,\gamma_{l',k'}^{b}\!\right)^{T}\!\left(\!\gamma_{l,k}^{b}\,-\,\gamma_{l',k'}^{b}\!\right)\!\right\rbrace \!f_{b}, l'\!\neq\! l \end{aligned}\  $$

In these formulations, the terms within the brackets can be readily computed from the training data. In other words, we can rewrite $V_{with}^{b}={f_{b}^{T}} U_{with}^{b}f_{b}\phantom {\dot {i}\!}$ and $V_{betw}^{b}={f_{b}^{T}} U_{betw}^{b}f_{b}\phantom {\dot {i}\!}$, in which $U_{with}^{b}\phantom {\dot {i}\!}$ and $U_{betw}^{b}\phantom {\dot {i}\!}$ are obtained from the feature vectors {*x*_*b*_(*n*)} and the unknown *f*_*b*_ is isolated. Here $U_{with}^{b}\phantom {\dot {i}\!}$ and $U_{betw}^{b}\phantom {\dot {i}\!}$ are square matrices with dimension *D*×*D*.

By optimizing *f*_*b*_, we expect to maximize the between-class difference and minimize the within-class variation. This is formulated as:

8 $$ \operatorname*{argmax}_{f_{b}} \frac{{f_{b}^{T}} U_{betw}^{b}\,f_{b}}{{f_{b}^{T}} U_{with}^{b}\,f_{b}}  $$

The kernel *f*_*b*_ is then derived by solving the generalized eigenvector problem:

9 $$ U_{betw}^{b}\,f_{b} = \lambda U_{with}^{b}\,f_{b}  $$

where *λ* is the generalized eigenvalue.

With the learned kernels {*f*_*b*_:*b*=1,…,*B*}, we obtain the transformed vectors {*y*_*b*_:*b*=1,…,*B*}. Each vector *y*_*b*_ is then rescaled so that its norm is the same as that of *x*_*b*_. In this way, the relative magnitudes and contributions of the various vectors in the transformed descriptor *y* remain unchanged when compared to the original descriptor *x*. The transformed descriptor *y* is finally obtained by concatenating the rescaled vectors. Based on these transformed descriptors, a linear-kernel multi-class SVM classifier is trained and then applied to classify the testing data.

### IICBU database and implementation

We used the publicly available IICBU 2008 database for our experiments. This database contains 11 separate datasets of different bioimage classification problems, and represents a broad range of real-life biological problems identified by experimental biologists. Each dataset features organelles, cells, or tissues, and includes noisy and unusual images typically present in biological databases. Excellent classification performance with near 100% accuracy has been reported for three datasets. We thus focused on the other eight datasets. Table 1 lists the main properties of the eight datasets. Example images are shown in Fig. 1. The lymphoma, liver gender and liver aging datasets provide color images. We converted them into grayscale using the standard RGB to grayscale conversion. For datasets with large intensity ranges, we rescaled the image intensities linearly to the range of 0 to 255, before extracting the visual descriptors.

For each dataset, we used five-fold cross validation for training and testing; and ten splits were used to measure the overall classification accuracy. For feature extraction and subcategory generation, we performed initial study on the terminal bulb aging dataset and experimented with parameter settings that were commonly used in related literature. The chosen parameter settings for this dataset were then used for all the datasets to maintain a common approach across datasets. The values of parameters have been described in the previous sections on visual descriptor extraction and subcategory generation. For SDT, the only parameter is the size *D* of the convolution kernel *f*_*b*_. The kernels applied to different feature modalities can have different sizes, hence there are a total of five size parameters to select. To do this, we specified that *D*∈[3,9], and employed a sequential search approach by fixing four size parameters while selecting the best setting for the fifth one. The sequential search was conducted on the training data, by performing multiple runs of four-fold cross validation within the training set and choosing the parameter *D* based on majority voting from these runs.

## Results and discussion

### Classification results using various descriptors

**Fig. 4:**
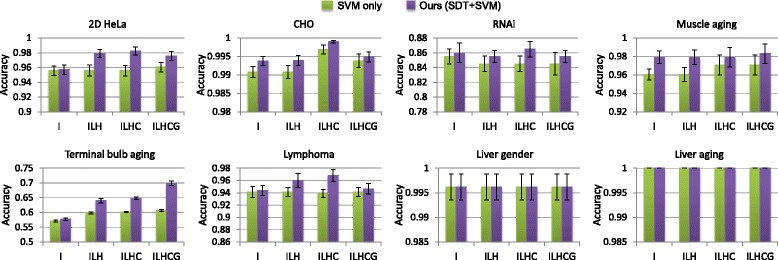
Classification results comparing various combinations of visual descriptors. Results are obtained by classifying the descriptors using (1) SVM only without SDT and (2) our method that applies SDT before SVM classification. The feature names IFV, LBP, HOG, CENTRIST and GIST are shortened as I, L, H, C and G, respectively. The mean accuracy and standard error are shown

Figure 4 shows the classification results with different combinations of visual descriptors using (i) linear-kernel SVM without the SDT component and (ii) our method with SDT before performing the SVM classification. The results show that the different approaches provided the same results for the liver gender and aging datasets, with saturated classification performance at 99.6% and 100%. For the other six datasets, our method outperformed the SVM only approach consistently when different feature descriptors were used. This demonstrates the advantage of our proposed SDT algorithm.

Overall, the results suggest that the ILHC descriptor should be used for most datasets (2D HeLa, CHO, RNAi, lymphoma, liver gender and liver aging); and ILHCG, which includes the additional GIST feature, is more effective for the muscle aging and terminal bulb aging datasets. The GIST feature describes the dominant spatial structure of an image. It provides different perspectives of an image compared to IFV, LBP and HOG, which summarizes the local visual information with varying encodings. It is also different from CENTRIST, which captures the global shape information. It can be seen from Fig. 1 that there is little spatial structural information in the 2D HeLa, CHO, RNAi, lymphoma, liver gender and liver aging datasets. Adding GIST could thus introduce noisy data and reduce the discriminative power of feature descriptors. However, for the muscle aging and terminal bulb aging datasets, the spatial distribution of tissues is an important clue for classification, and hence the ILHCG descriptor is more suitable.

**Fig. 5:**
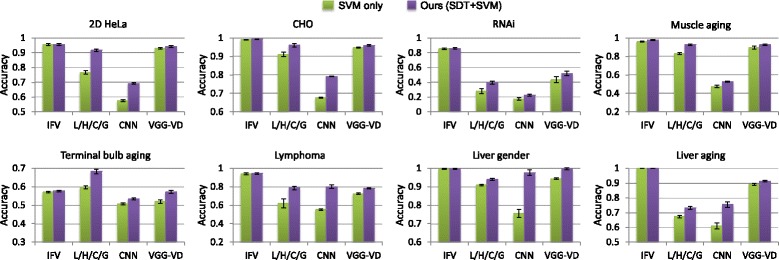
Classification results comparing different descriptors. Results are obtained by classifying the descriptors using (1) SVM only without SDT and (2) our method that applies SDT before SVM classification. The features tested include IFV, L/H/C/G (denoting the combination of LBP, HOG, CENTRIST and GIST), features generated using the VGG-VD model pretrained on ImageNet, and features generated using a CNN model trained on each dataset. The mean accuracy and standard error are shown

Figure 5 shows that IFV was the most critical descriptor that contributed to the overall high classification accuracy. The classification performance was much lower if using only the other four texture features (LBP, HOG, CENTRIST and GIST, namely L/H/C/G), except for the terminal bulb aging. The L/H/C/G feature was particularly ineffective for the lymphoma, RNAi and liver aging datasets. A major difference of IFV from L/H/C/G is that IFV creates a mid-level feature representation with Fisher encoding of multi-scale local SIFT features, based on unsupervised learning of GMM distributions for each dimension of the local features. Such an encoding effectively exploits the feature space characteristics of the whole dataset and provides a more comprehensive feature representation than the other features. For the terminal bulb aging dataset, better results were obtained using L/H/C/G than IFV. We suggest that this is because the global spatial and structural information in the terminal bulb images is important for distinguishing the different ages. The LBP, HOG and GIST descriptors explicitly encode such information by concatenating cell-level features, and these additional information helps to enhance the descriptiveness of textures by these descriptors.

The high discriminative power of IFV also means that IFV is already highly optimized. As a result, the improvement by SDT on IFV descriptors was relatively low. It can be seen from Fig. 4 that with IFV features, less than 1% improvement in classification accuracy was typically obtained when SDT was applied. However, the improvement by SDT on the multi-modal descriptor was apparent. For example, on the RNAi and lymphoma datasets, the ILHC descriptor was the least effective when classified using SVM only, but generated the highest classification performance when SDT was applied. On the 2D HeLa and terminal bulb aging datasets, the improvement from SDT was minimal when only IFV was used, whereas the improvement was much larger with the ILHC or ILHCG descriptor. We found that this was because the improvement by SDT on the LBP, HOG, CENTRIST and GIST features was much higher (3 to 16%) than that on the IFV feature (less than 1%). Since IFV is a learning-based encoding of local descriptors and the other features are computed based on predefined rules, our subcategory-based discriminative learning method could provide much greater enhancement of the other texture features than the IFV feature. These improvements led to the overall improved classification results using the ILHC or ILHCG descriptors. For illustration, we show the results using L/H/C/G (the combination of LBP, HOG, CENTRIST and GIST) in Fig. 5; but similar improvements were obtained for subsets of L/H/C/G.

We also conducted paired t-test to verify the statistical significance of improvement by SDT for different descriptor choices. The classification outputs using SVM classification only were compared with the outputs using our method, and the null hypothesis was that both approaches would provide the same results. With IFV as the feature descriptor, the *p*-value was around 0.1 for all datasets (except liver gender and aging), indicating that the improvement by SDT on IFV descriptors was not statistically significant. However, using the L/H/C/G descriptors, the improvement was statistically significant on all datasets, with *p*-values less than 0.05 (in the range of 0.001 to 1*e*−20). Using the ILHC or ILHCG descriptors, the *p*-values for the 2D HeLa, CHO, terminal bulb aging, and lymphoma datasets were all less than 0.05 (in the range of 0.03 to 2*e*−9), while the *p*-values for the RNAi and muscle aging datasets were around 0.1. For the muscle aging dataset, we suggest the insignificant improvement was mainly because the classification performance using SVM only was already very high and there was limited scope of improvement for SDT. For the RNAi dataset, even with the high degree of improvement by SDT on the L/H/C/G descriptors, the classification performance by the L/H/C/G descriptors was still low, and hence such improvement could only boost the final classification results slightly. Also, in the RNAi dataset, each image class contains only 20 images. This small amount of images would limit the learning capability of SDT, and this could be an important factor affecting the degree of improvement provided by SDT on this dataset.

For further evaluation, we tested two deep learning approaches with convolutional neural network (CNN) to generate the feature descriptors, which have recently become popular in biomedical imaging analysis. First, we applied the very deep VGG-VD model [51] that was pretrained on ImageNet. We chose the VGG-VD model since it reported the state-of-the-art performance on multiple benchmarks in general computer vision. Second, we trained a CNN model for each dataset. The well-known AlexNet [52] was adopted as the base network, with two middle convolutional layers removed to reduce the network complexity to better accommodate the much smaller number of images in our datasets compared to ImageNet; and affine transformation was used for data augmentation. In both cases, the 4096-dimensional descriptor from the last fully connected layer was used as the image descriptor.

Figure 5 shows that the CNN features (based on models trained for each dataset) resulted in the lowest performance in most cases. While we tried several variations of the AlexNet architecture, similarly low classification performance was obtained. We suggest that this was due to the insufficient number of images available to train the CNN model. Although it would be possible to further enhance the CNN models, such experiments were beyond our scope of this study. Also note that when classification output from CNN was directly used, the classification results were 4 to 15% lower than using the 4096-dimension descriptor with SVM. This indicates the advantage of using SVM in place of the softmax layer for the trained models. The VGG-VD features performed generally better than L/H/C/G except for the terminal bulb aging dataset, implying that the pretrained ImageNet model was transferable to the IICBU datasets. The IFV features were more effective than VGG-VD, and this demonstrates the advantage of the Fisher encoding of local descriptors. It can also be seen that our SDT method provided the largest improvement when CNN features were used. This suggests that when the descriptors are less descriptive, there is larger scope of enhancement by SDT and the optimization process in SDT could better transform the feature space to reduce the intra-class variation and increase the inter-class difference. The improvement by SDT on CNN and VGG-VD features was statistically significant for all datasets, with *p*-values in the range of 0.003 to 1*e*−20. This demonstrates that our SDT algorithm can be useful to deep learning features, and hence can be easily applied to other problem domains in biomedical or general imaging.

In addition, we also tested various other features, including the more traditional encoding techniques of local SIFT descriptors (i.e. bag-of-features (BOF) and LLC), and SURF, gray level co-occurrence matrix (GLCM) and wavelet features. We found that IFV was more effective than the BOF or LLC encoded SIFT descriptors, with on average 15% improvement in classification accuracy. Inclusion of SURF, GLCM or wavelet features degraded the classification performance by 3 – 10% compared to using LBP, HOG, GIST and CENTRIST.

### Comparison with existing studies

Table 2 shows our classification results in comparison with the existing studies. We obtained better results over the state-of-the-art for six of the eight datasets (except terminal bulb aging and RNAi) for both ILHC and ILHCG descriptors. In addition, among the compared approaches, three approaches [8, 9, 13] focused on performing general bioimage classification for different problem domains. Our method was also designed to support general bioimage classification without involving domain-specific processing, and achieved much higher accuracy over these approaches for all datasets. Furthermore, from Fig. 4 and Table 2, we can see that even with SVM only, the best performing descriptor provided better classification accuracy over the state-of-the-art for six datasets (except RNAi and terminal bulb aging). For example, using ILHCG and SVM classification without SDT, we obtained 96.1% classification accuracy, compared to 94.4% of the state-of-the-art [13, 15] for the 2D HeLa dataset. These results further demonstrate the effectiveness of our proposed visual descriptor.

**Table 2:** Classification accuracies (%) compared to the existing studies

Dataset	Ours-ILHCG	Ours-ILHC	[9]	[8]	[13]	[15]	[11]	[10]	[23]	[14]
2D HeLa	**97.6** ±**1.4**	**98.3** ±**1.2**	84	68.3	94.4	94.4	90.7	–	–	–
CHO	**99.5** ±**0.4**	**99.9** ±**0.1**	93	93.1	98.5	99.4	98.4	–	–	–
RNAi	85.5 ±2.5	86.5 ±3.3	82	55.0	67.5	**92.0**	90.1	–	–	–
Muscle aging	**98.3** ±**2.7**	**97.9** ±**3.6**	53	89.6	–	–	–	–	–	–
Terminal bulb aging	69.5 ±2.4	64.8 ±2.4	49	51.1	44.6	–	–	–	–	**69.9**
Lymphoma	**94.6** ±**2.7**	**96.8** ±**3.1**	85	70.9	–	–	–	92.7	63.3	–
Liver gender	**99.6** ±**0.8**	**99.6** ±**0.8**	69	91.7	–	–	–	99.2	97.3	–
Liver aging	**100.0** ±**0.0**	**100.0** ±**0.0**	51	73.8	–	–	–	96.4	–	–

Boldface indicates the best result on the individual dataset

**Fig. 6:**
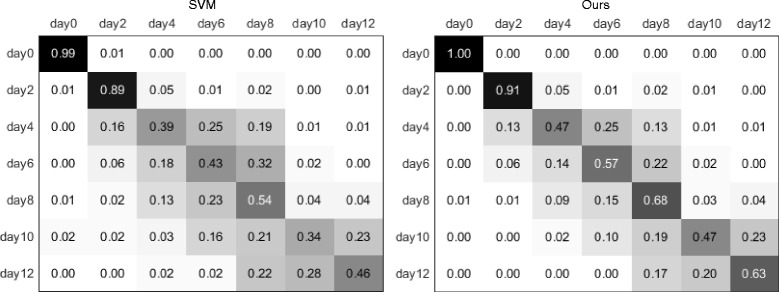
Results on the terminal bulb aging dataset. Confusion matrices of the classification results using SVM only and our method (SDT+SVM) are shown

**Fig. 7:**
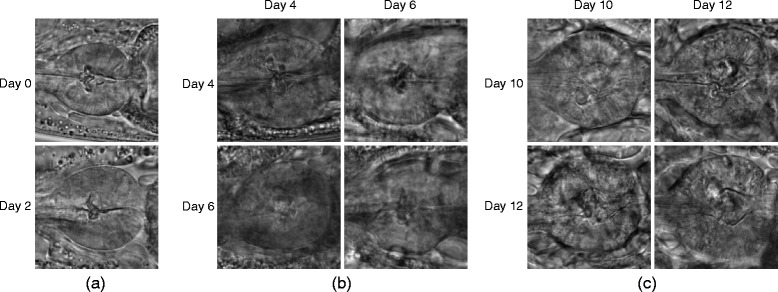
Visual results on the terminal bulb aging dataset. The samples show **a** images of class ‘day 0’ and ‘day 2’ that are correctly classified; **b** cases where class ‘day 4’ (*top row*) or ‘day 6’ (*bottom row*) are classified as ‘day 4’ (*left column*) or ‘day 6’ (*right column*); and **c** cases where class ‘day 10’ (*top row*) or ‘day 12’ (*bottom row*) are classified as ‘day 10’ (*left column*) or ‘day 12’ (*right column*). In **b** and **c**, the two off-diagonal images illustrate the misclassification cases

For the terminal bulb aging dataset, our classification result was 0.4% lower than the state-of-the-art [14]. The approach [14] involves a multi-level classification model with additional middle classes. It is however unknown how this approach would perform for the other datasets. To further analyze our method performance, we generated the confusion matrices using the SVM only approach and our method, as shown in Fig. 6. A comparison between the two confusion matrices shows that our method obtained consistent improvement for each class over the SVM only results. The first two classes were easily differentiated from the other classes with distinct structural patterns in the tissues (examples shown in Fig. 7 a. For the other five classes, we observed that the images become more blurred as the terminal bulb ages, and it becomes more difficult to distinguish the different ages. Figure 7 shows examples of misclassification between days 4 and 6, and between days 10 and 12. It can be seen that the visual characteristics in these images are hard to differentiate between different classes, and images of the same class show varying characteristics as well. It would thus be challenging to obtain a feature representation to effectively classify the images. Furthermore, we found that including more spatial information into the feature representation, such as by extracting extra texture features from the middle region of the images, could improve the classification performance. This would be our future study as a customized design for the terminal bulb aging classification.

**Fig. 8:**
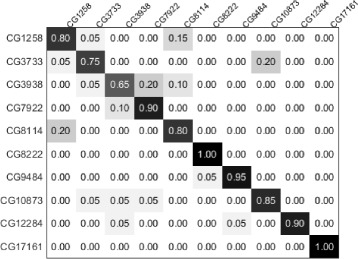
Results on the RNAi dataset. Confusion matrix of the classification results is shown

**Fig. 9:**
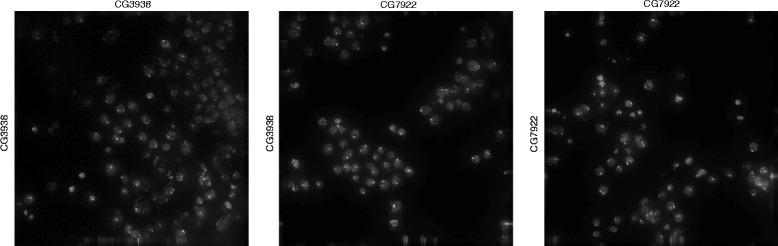
Visual results on the RNAi dataset. The *left* and *middle images* are of class ‘CG3938’ and the *right image* is of class ‘CG7922’. The *left* and *right images* are correctly classified, but the *middle image* is misclassified as class ‘CG7922’

For the RNAi dataset, the compared approaches [11, 15] outperformed our method. In particular, the approach [15] included a shape-based feature design based on the GLCM representation, and provided the state-of-the-art classification result with 5.5% advantage over our method. However, the approaches [11, 15] were less effective for the 2D HeLa and CHO datasets and hence less generalizable when compared to our method. We suggest that the main uniqueness of the RNAi dataset is that the images contain many well isolated cells and the spatial structure is not informative when classifying the images. These characteristics thus limited the effectiveness of our feature descriptor for the RNAi dataset. Figure 8 shows the confusion matrix obtained using our method. The lowest classification accuracy was observed for the class of gene CG3938, and 20% of it was misclassified as CG7922. Example of misclassification is shown in Fig. 9. It can be seen that while the two genes have different functionalities, i.e. regulation of cell cycles or DNA repair, they show similar visual characteristics and could be easily confused to the untrained eyes. Based on these comparisons, we suggest that a customized feature design at the individual cell-level could be investigated for further improving the classification performance of the RNAi dataset.

It is worth noting that in our experiments we conducted ten runs of five-fold cross validation, while the compared approaches used different cross validation techniques, as summarized in Table 3. For example, the approach [14] used a single three-fold cross validation. As a result of the difference in evaluation protocol, their performance advantage of 0.4% on the terminal bulb aging dataset might not be conclusive.

**Table 3:** Evaluation protocol of compared approaches

	Ours	[9]	[8]	[13]	[15]	[11]	[10]	[23]	[14]
# Folds in cross validation	5	-	5	10	5	5	-	-	3
# Splits	10	1	5	1	1	100	1	1	1

### Comparison with dimension reduction

While our SDT method does not involve dimension reduction, it adds an additional step prior to the SVM classification, and has some similarity with LDA that the transform tries to reduce the within-class variation and increase the between-class difference based on discriminative learning. In addition, considering the small number of images in the datasets, dimension reduction could be useful to address the over-fitting issue. We compared our method with several popular discriminative dimension reduction techniques: LDA, GDA [48], FML [43], and FDDL [46]. GDA is a kernelized version of LDA. FML is based on distance metric learning and demonstrates good performance when coupled with IFV and SVM [45]. FDDL, being a supervised dictionary learning technique with Fisher discrimination constraints, can be used for dimension reduction as well. To have direct comparison with SDT, the dimension reduced descriptors from LDA, GDA and FML were classified using linear-kernel SVM. For FDDL, we used the integrated sparse representation-based classification technique. The parameters used in these approaches were tuned manually. We also performed the paired t-test to verify the statistical significance of improvement of SDT over LDA, GDA, FDDL and FML.

**Fig. 10:**
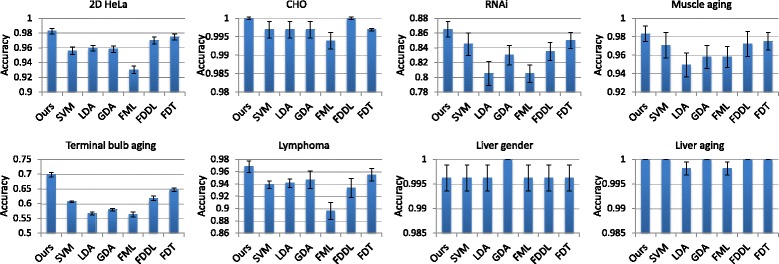
Classification results comparing our method with the other related techniques. Results are obtained using our method (SDT+SVM), SVM only classification, the discriminative dimension reduction techniques (LDA, GDA, FML and FDDL), and FDT that replaces the subcategory-based computation in SDT with a class-based formulation. The mean accuracy and standard error are shown

As shown in Fig. 10, when the classification accuracies were near 100% on the CHO, liver gender and aging datasets, our method performed similarly to GDA and FDDL. The improvement provided by our method is more clearly observed for the other five datasets with non-saturated classification accuracies. Compared to the SVM only approach, FDDL and GDA resulted in slightly higher accuracies for some datasets while LDA and FML often affected the results negatively. The advantage of SDT over LDA, GDA and FML was statistically significant with *p*-values less than 0.05 for all five datasets. The improvement of SDT over FDDL was statistically significant for the 2D HeLa, terminal bulb aging and lymphoma datasets. For the RNAi and muscle aging datasets, FDDL performed similarly to SVM, and the improvement of SDT over FDDL on these datasets was insignificant. To explain this insignificance, our previous discussion regarding the improvement of SDT over SVM can be applied here as well. Overall, these results demonstrate that it is not advantageous to apply dimension reduction techniques on the high-dimensional descriptor before SVM classification. Compared to the commonly used dimension reduction techniques, our SDT method could retain and enhance the discriminative information of the descriptors to a larger extent. Therefore, it is generally more effective by first transforming the feature descriptors with our SDT model before SVM classification.

### Use of subcategories

In SDT, the within- and between-class variations are computed based on subcategories. To analyze the effect of such a subcategory-based model, we compared our results with an alternative approach that replaces the subcategory-level computation by class-level computation. We name this alternative approach Fisher discriminant transform (FDT). Briefly, we change Eqs. (1) and (2) to measure the within- and between-class variations similarly to the standard Fisher discrimination, by computing the distance between data samples and class means, and between class means and the overall mean. This transform also keeps the descriptor dimension unchanged. The subcategory information is not used in FDT, hence result comparison between FDT and our SDT method would indicate the use of subcategories in the feature transform.

Figure 10 shows the evaluation results. It can be seen that FDT generally outperformed all the compared approaches, indicating the benefit of discriminative descriptor transform via a block-wise convolution-based design. In addition, our subcategory-based method shows higher classification accuracies than FDT consistently for all datasets. In general imaging, it is generally considered that sub-categorization methods could better accommodate the feature space variation, and encouraging performance has been reported recently [35, 53–55]. Our comparison with FDT confirms the advantage of the subcategory-based feature space modeling over the usual class-level representation.

Regarding the clustering method for generating the subcategories, we chose the LSC algorithm mainly due to its efficiency. For example, on the smallest RNAi dataset of 200 images, LSC clustering took about 0.4 seconds while the well-known SSC method took about 50 minutes. SSC is similar to LSC except that SSC used the standard Lasso for sparse representation while LSC used the highly efficient LLC algorithm. Such a long computation time prevented us from using SSC for our current study. In addition, *k*-means clustering is also quite efficient, requiring about 0.6 seconds for the RNAi dataset. However, the classification performance using *k*-means clustering was on average 0.5% lower than that using LSC clustering for all datasets.

### Further analysis of SDT

To further elaborate the effect of our SDT method, we provide analysis of the intermediate results when applying the method. The underlying objective of SDT is to minimize the within-class and maximize the between-class differences. We thus measured the within- and between-class differences before and after applying SDT, and the margins between separation hyperplanes from the trained SVMs correspondingly. These measures help to demonstrate the use of SDT intuitively.

**Fig. 11:**
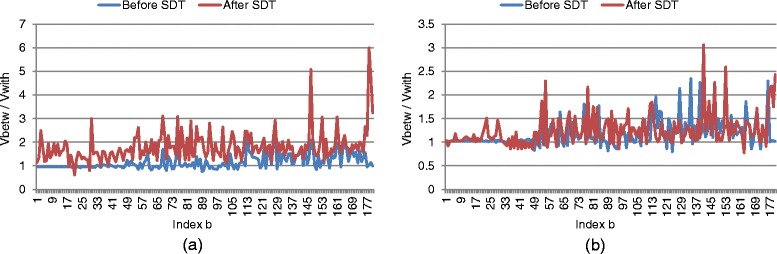
Effects of SDT on feature space distribution. Ratios between $V_{betw}^{b}$ and $V_{with}^{b}$ are computed before and after applying SDT, from **a** the training set and **b** the testing set of the terminal bulb aging dataset

Recall that our ILHCG descriptor *x* is partitioned into *B* blocks of feature vectors *x*={*x*_*b*_:*b*=1,…,*B*} with *B*=180, and the within- and between-class differences $V_{betw}^{b}\phantom {\dot {i}\!}$ and $V_{with}^{b}\phantom {\dot {i}\!}$ are derived for each vector *x*_*b*_ separately. We computed the ratio between $V_{betw}^{b}\phantom {\dot {i}\!}$ and $V_{with}^{b}\phantom {\dot {i}\!}$ for each *x*_*b*_, and concatenated the ratios together as the overall measurement of the feature space distribution. Figure 11 a shows the ratios obtained before and after applying SDT on the training set of the terminal bulb aging dataset. It can be seen that the ratios became higher after SDT was applied, indicating larger between-class difference compared to within-class variation. Such an observation is aligned with the optimization objective of SDT in Eq. (8). It was also found that in the original feature space, the separation margin between hyperplanes from the trained SVM was on average 0.14. In the transformed feature space after applying SDT, the separation margin became 0.19. Therefore, with SDT the transformed features could be better separated between different classes and more accurate classification became possible.

On the other hand, an important aspect is if the effect of SDT shown on the training set is transferable to the testing set. To analyze this, we computed the ratios on the testing set. As shown in Fig. 11 b, among the 180 ratios corresponding to 180 blocks of feature vectors, 130 ratios became higher after applying SDT. This helps to explain the higher classification performance using our method compared to using only SVM without SDT as shown in Fig. 4. The overall increase in ratios after applying SDT was lower than those on the training set. This is because similar to the other discriminative learning techniques (e.g. SVM), the testing set typically exhibits some different feature space characteristics from the training set. Such differences would subsequently affect the effectiveness of the learned model when applied to the testing set.

In addition, as a feature transform method, SDT can be considered related to the artificial neural network (ANN). Specifically, in SDT, the learned kernels are convoluted with the feature vectors to generate the transformed descriptor; and in ANN, the outputs of hidden layers are transformed descriptors with the weights optimized based on the classification objective. The main difference of SDT from ANN is that our optimization objective is constructed based on the intra- and inter-class variations derived from subcategories and SDT is applied to each block of feature vectors rather than the whole feature descriptors.

To compare our optimization method with ANN in a fair manner, we designed the following ANN-based approach. First, since in our method we learned 180 convolution kernels for the 180 blocks of feature vectors, we trained 180 ANNs correspondingly as well. Each ANN was configured to have an input layer for the feature vectors, one hidden layer as the feature transform layer, and one output layer for classification. The hidden layer had the same number of nodes as the input feature vector, so that the feature dimension remained unchanged after this layer, simulating the effect of our SDT-based feature transform. After training the 180 ANNs, the transformed features generated at the hidden layer were then concatenated together to produce the transformed descriptors, which were then used to learn an SVM classifier. For a testing image, the transformed descriptor was derived from the learned ANNs and then classified using the SVM.

The results show that our SDT method outperformed this ANN-based method on six datasets, with improvement in classification accuracy of 1.8% (2D HeLa), 0.3% (CHO), 1.5% (RNAi), 0.8% (muscle aging), 7.7% (terminal bulb aging), and 3.0% (lymphoma). The same results were obtained on the liver gender and aging datasets. Also, the ANN-based method outperformed the SVM only classification and dimension reduction techniques with 0.5 to 2.0% improvement for five datasets (except CHO, liver gender and aging), while we found that a standard ANN classification (using 3-layer ANN to classify the high-dimension feature descriptors directly) underperformed the SVM only classification by 0.3 to 9%. This implies the advantage of having block-wise feature transform and the additional SVM classification of the transformed descriptors. We suggest that it could be possible to further enhance the ANN-based method with convolutional layers, and this would be an interesting direction in our future study.

## Conclusions

In this paper, we present a general method for bioimage classification. The images are represented with highly discriminative high-dimensional visual descriptors comprising multi-modal features. A subcategory discriminant transform (SDT) method is designed to further enhance the discriminative power of the descriptors using block-wise convolution processing, in which the convolution kernels are learned based on subcategory-level discriminative modeling. We have evaluated our method on the publicly available IICBU 2008 database, which contains eight individual datasets of various types of microscopy images and different multi-class classification problems. Our method outperformed state-of-the-art approaches for six datasets.

## References

[1] Peng H (2008). Bioimage informatics: a new area of engineering biology. Bioinformatics.

[2] Eliceiri KW, Berthold MR, Goldberg IG, Ibanez L, Manjunath BS, Martone ME, Murphy RF, Peng H, Plant AL, Roysam B, Stuurmann N, Swedlow JR, Tomancak P, Carpenter AE (2012). Biological imaging software tools. Nat Methods.

[3] Carpenter AE, Jones TR, Lamprecht MR, Clarke C, Kang IH, Friman O, Guertin DA, Chang JH, Lindquist RA, Moffat J, Golland P, Sabatini DM (2006). CellProfiler: image analysis software for identifying and quantifying cell phenotypes. Genome Biol..

[4] Shamir L, Orlov N, Eckley DM, Macura TJ, Goldberg IG (2008). IICBU 2008: a proposed benchmark suite for biological image analysis. Med Biol Eng. Comput..

[5] Misselwitz B, Strittmatter G, Periaswamy B, Schlumberger MC, Rout S, Horvath P, Kozak K, Hardt WD (2010). Enhanced CellClassifier: a multi-class classification tool for microscopy images. BMC Bioinformatics.

[6] Pau G, Fuchs F, Sklyar O, Boutros M, Huber W (2010). EBImage - an R package for image processing with applications to cellular phenotypes. Bioinformatics.

[7] Held M, Schmitz MH, Fischer B, Walter T, Neumann B, Olma MH, Peter M, Ellenberg J, Gerlich DW (2010). CellCognition: time-resolved phenotype annotation in high-throughput live cell imaging. Nat Methods.

[8] Zhou J, Lamichhane S, Sterne G, Ye B, Peng H (2013). BIOCAT: a pattern recognition platform for customizable biological image classification and annotation. BMC Bioinformatics.

[9] Shamir L, Orlov N, Eckley DM, Macura TJ, Johnston J, Goldberg IG (2008). Wndchrm - an open source utility for biological image analysis. Source Code Biol Med..

[10] Meng T, Lin L, Shyu M, Chen S. Histology image classification using supervised classification and multimodal fusion. IEEE Int Symp Multimedia. 2010;:145–52.

[11] Zhang B, Pham TD (2011). Phenotype recognition with combined features and random subspace classifier ensemble. BMC Bioinformatics.

[12] Song Y, Cai W, Huang H, Wang Y, Feng D, Chen M (2013). Region-based progressive localization of cell nuclei in microscopic images with data adaptive modeling. BMC Bioinformatics.

[13] Coelho LP, Kangas JD, Naik AW, Osuna-Highley E, Glory-Afshar E, Fuhrman M, Simha R, Berget PB, Jarvik JW, Murphy RF (2013). Determining the subcellular location of new proteins from microscope images using local features. Bioinformatics.

[14] Meng T, Shyu M. Biological image temporal stage classification via multi-layer model collaboration. IEEE Int Symp Multimedia. 2013;:30–7.

[15] Nanni L, Brahnam S, Ghidoni S, Menegatti E, Barrier T (2013). A comparison of methods for extracting information from the co-occurrence matrix for subcellular classification. Expert Syst Appl.

[16] Tahir M, Khan A, Majid A, Lumini A (2013). Subcellular localization using fluorescence imagery: utilizing ensemble classification with diverse feature extraction strategies and data balancing. Appl Soft Comput.

[17] Xu Y, Zhu J, Chang EI, Lai M, Tu Z (2014). Weakly supervised histopathology cancer image segmentation and classification. Med Image Anal..

[18] Yang F, Xu Y, Wang S, Shen H (2014). Image-based classification of protein subcellular location patterns in human reproductive tissue by ensemble learning global and local features. Neurocomputing.

[19] Abbas SS, Dijkstra TMH, Heskes T (2014). A comparative study of cell classifiers for image-based high-throughput screening. BMC Bioinformatics.

[20] Jiang M, Zhang S, Huang J, Yang L, Metaxas DN (2015). Joint kernel-based supervised hashing for scalable histopathological image analysis. Int Conference Med Image Comput Comput Assisted Intervention, Lecture Notes Comput Sci.

[21] Su H, Xing F, Kong X, Xie Y, Zhang S, Yang L. Robust cell detection and segmentation in histopathological images using sparse reconstruction and stacked denoising autoencoders. MICCAI. 2015;:383–90. 10.1007/978-3-319-24574-4_46 PMC5081214 27796013

[22] Tabesh A, Teverovskiy M, Pang HY, Kumar V, Verbel D, Kotsianti A, Saidi O (2007). Multifeature prostate cancer diagnosis and gleason grading of histological images. IEEE Trans Med Imag..

[23] Herve N, Servais A, Thervet E, Olivo-Marin J, Meas-Yedid V. Statistical color texture descriptors for histological image analysis. ISBI. 2011;:724–7.

[24] Sparks R, Madabhushi A (2013). Explicit shape descriptors: novel morphologic features for histopathology classification. Med Image Anal..

[25] Kandemir M, Zhang C, Hamprecht FA (2014). Empowering multiple instance histopathology cancer diagnosis by cell graphs. Int Conference Med Image Comput Comput Assisted Intervention, Lecture Notes Comput Sci.

[26] Peter L, Pauly O, Chatelain P, Mateus D, Navab N (2015). Scale-adaptive forest training via an efficient feature sampling scheme. Int Conference Med Image Comput Comput Assisted Intervention, Lecture Notes Comput Sci.

[27] Xu X, Lin F, Ng C, Leong KP (2015). Adaptive co-occurrence differential texton space for HEp-2 cell classification. Int Conference Med Image Comput Comput Assisted Intervention, Lecture Notes Comput Sci.

[28] Barker J, Hoogi A, Depeursinge A, Rubin DL (2016). Automated classification of brain tumor type in whole-slide digital pathology images using local representative tiles. Med Image Anal.

[29] Zhou Y, Chang H, Barner K, Spellman P, Parvin B. Classification of histology sections via multispectral convolutional sparse coding. IEEE Conference Comput Vision Pattern Recognit. 2014;:3081–8. 10.1109/CVPR.2014.394 PMC4279924 25554749

[30] Otalora S, Cruz-Roa A, Arevalo J, Atzori M, Madabhushi A, Judkins AR, Gonzalez F, Muller H, Depeursinge A (2015). Combining unsupervised feature learning and riesz wavelets for histopathology image representation: application to identifying anaplastic medulloblastoma. Int Conference Med Image Comput Comput Assisted Intervention, Lecture Notes Comput Sci.

[31] BenTaieb A, Li-Chang H, Huntsman D, Hamarneh G (2015). Automatic diagnosis of ovarian carcinomas via sparse multiresolution tissue representation. Int Conference Med Image Comput Comput Assisted Intervention, Lecture Notes Comput Sci.

[32] Vu TH, Mousavi HS, Monga V, Rao G, Rao A, IEEE Trans Med Imag. Histopathological image classification using discriminative feature-oriented dictionary learning. 2016; 35(3):738–51. 10.1109/TMI.2015.2493530 PMC4807738 26513781

[33] Perronnin F, Sanchez J, Mensink T (2010). Improving the fisher kernel for large-scale image classification. Eur Conference Comput Vision, Lecture Notes Comput Sci.

[34] Cimpoi M, Maji S, Kokkinos I, Mohamed S, Vedaldi A. Describing textures in the wild. IEEE Conference Comput Vision Pattern Recognit. 2014;:3606–13.

[35] Song Y, Cai W, Li Q, Zhang F, Feng D, Huang H. Fusing subcategory probabilities for texture classification. IEEE Conference Comput Vision Pattern Recognit. 2015;:4409–17.

[36] Ojala T, Pietikainen M, Maenpaa T (2002). Multiresolution gray-scale and rotation invariant texture classification with local binary patterns. IEEE Trans Pattern Anal Mach Intell..

[37] Felzenszwalb PF, Girshick RB, McAllester D, Ramanan D (2010). Object detection with discriminatively trained part-based models. IEEE Trans Pattern Anal Mach Intell..

[38] Oliva A, Torralba A (2001). Modeling the shape of the scene: a holistic representation of the spatial envelope. Int J Comput Vis.

[39] Wu J, Rehg JM (2011). CENTRIST: a visual descriptor for scene categorization. IEEE Trans Pattern Anal Mach Intell..

[40] Chatfield K, Lempitsky V, Vedaldi A, Zisserman A. The devil is in the details: an evaluation of recent feature encoding methods. Br Machine Vision Conf. 2011;:1–12.

[41] Chen D, Cao X, Wen F, Sun J. Blessing of dimensionality: high-dimensional feature and its efficient compression for face verification. IEEE Conference Comput Vision Pattern Recognit. 2013;:3025–2.

[42] Zhang Y, Wu J, Cai J. Compact representation for image classification: to choose or to compress?IEEE Conference Comput Vision Pattern Recognit. 2014;:907–14.

[43] Simonyan K, Parkhi OM, Vedaldi A, Zisserman A. Fisher vector faces in the wild. Br Machine Vision Conf. 2013;:1–12.

[44] Ren X, Ramanan D. Histograms of sparse codes for object detection. Br Machine Vision Conf. 2013;:3246–53.

[45] Parkhi OM, Simonyan K, Vedaldi A, Zisserman A. A compact and discriminative face track descriptor. IEEE Conference Comput Vision Pattern Recognit. 2014;:1693–700.

[46] Yang M, Zhang L, Feng X, Zhang D. Fisher discrimination dictionary learning for sparse representation. IEEE Int Conference Comput Vision. 2011;:543–50.

[47] Yang M, Dai D, Shen L, Gool LV. Latent dictionary learning for sparse representation based classification. IEEE Conference Comput Vision Pattern Recognit. 2014;:4138–45.

[48] Baudat G, Anouar F (2000). Generalized discriminant analysis using a kernel approach. Neural Comput.

[49] Wang J, Yang J, Yu K, Lv F, Huang T, Gong Y. Locality-constrained linear coding for image classification. IEEE Conference Comput Vision Pattern Recognit. 2010;:3360–7.

[50] Elhamifar E, Vidal R. Sparse subspace clustering. IEEE Conference Comput Vision Pattern Recognit. 2009;:2790–7.

[51] Cimpoi M, Maji S, Vedaldi A. Deep filter banks for texture recognition and segmentation. IEEE Conference Comput Vision Pattern Recognit. 2015;:3828–36. 10.1007/s11263-015-0872-3 PMC4946812 27471340

[52] Krizhevsky A, Sutskever I, Hinton GE. Imagenet classification with deep convolutional neural networks. Annu Conference Neural Inf Process Syst. 2012;:1–9.

[53] Divvala SK, Efros AA, Hebert M (2011). How important are “deformable parts” in the deformable parts model?. European Conference on Computer Vision, Lecture Notes in Computer Science.

[54] Dong J, Xia W, Chen Q, Feng J, Huang Z, Yan S. Subcategory-aware object classification. IEEE Conference Comput Vision Pattern Recognit. 2013;:827–34.

[55] Zhu X, Vondrick C, Ramanan D, Fowlkes C. Do we need more training data or better models for object detection?Br Machine Vision Conference. 2012;: pp. 1–11.

